# Network Pharmacology and In Vitro Experimental Validation Reveal the Anti-Inflammatory and Anti-Apoptotic Effects of Lotus Leaf Extract in Treating Inflammatory Diarrhea in Pigs

**DOI:** 10.3390/cimb47050314

**Published:** 2025-04-28

**Authors:** Yu Zheng, Jiana Zheng, Jiao Wang, Junxin Li, Jiali Liu, Bohan Zheng, Qinjin Li, Xiaohong Huang, Zhaoyan Lin

**Affiliations:** 1 College of Animal Science, Fujian Agriculture and Forestry University, Fuzhou 350002, China; zy931891704@163.com (Y.Z.); zhengjiana99@163.com (J.Z.); wj18438133218@163.com (J.W.); 18059732920@163.com (J.L.); 2021720930@yangtzeu.edu.cn (J.L.); zhengbohan@fafu.edu.cn (B.Z.); liqinjin0809@163.com (Q.L.); 2 University Key Laboratory for Integrated Chinese Traditional and Western Veterinary Medicine and Animal Healthcare in Fujian Province/Fujian Key Laboratory of Traditional Chinese Veterinary Medicine and Animal Health, Fujian Agriculture and Forestry University, Fuzhou 350002, China

**Keywords:** lotus leaf, inflammatory diarrhea in pigs, network pharmacology, molecular docking, apoptosis

## Abstract

The objective of this research was to investigate the efficacy of lotus leaf in the prevention and treatment of inflammatory diarrhea in pigs, utilizing network pharmacology and in vitro methodologies. Initially, LC-MS was employed to analyze the constituents of lotus leaf extract (LLE); then, the TCMSP database was utilized to identify the active components and their corresponding targets. The GeneCards database was consulted to identify disease-related targets pertinent to inflammatory diarrhea in pigs. A drug ingredient–disease target network was constructed using Cytoscape software. Subsequently, the STRING database facilitated protein interaction analysis, which was also visualized through Cytoscape. Gene Ontology (GO) functional annotation and Kyoto Encyclopedia of Genes and Genomes (KEGG) enrichment analysis were conducted based on the genes shared between disease and LLE targets. Molecular docking of the active ingredients with key targets was performed using Autodock Vina. Subsequently, an in vitro LPS-induced inflammation model was established using IPEC-J2 cells to validate the predictions made through network pharmacology. Verification was conducted via flow cytometry and Western blot analysis. The LC-MS assay and TCMSP retrieval results revealed that Quercetin, Nuciferine, Kaempferol, Leucodelphinidin, and Catechin were identified as the main compounds of LLE, associated with 181 potential targets. A total of 5995 targets were linked to inflammatory diarrhea in pigs, with 159 overlapping targets identified between the bioactive compounds and the disease. Notable key targets included TNF-α, IL-6, caspase-3, TP53, and AKT, which are integral to inflammation and apoptosis processes. GO functional annotation indicated significant enrichment in biological processes such as gene expression regulation and transcription from RNA polymerase II promoters. KEGG pathway analysis highlighted critical pathways, including TNF signaling and apoptosis. Furthermore, molecular docking analyses demonstrated that the bioactive components of lotus leaf exhibited a strong binding affinity for essential targets, including AKT1, BAX, caspase-3, CCL2, IL-6, IL-10, MPK14, NOS3, PTGS1, and TNF-α. In vitro experiments confirmed that LLE significantly inhibited LPS-induced apoptosis in IPEC-J2 cells and suppressed the activation of the TNF-α-mediated apoptosis pathway. This study offers novel insights into the therapeutic potential of Chinese medicine and its constituents in addressing inflammatory diarrhea in pigs.

## 1. Introduction

Diarrhea is a common clinical sign in the pig farming industry, primarily associated with difficulties or incomplete absorption of water in the large intestine [1]. Diarrhea has already brought about tremendous economic losses in pig farms worldwide [2].The disease can result from various factors, including viral or bacterial infections, nutritional malabsorption, and food poisoning, leading to weakened immunity in animals. Its characteristics include loose or watery stools, dehydration, and electrolyte imbalances [3]. It is worth noting that clinical cases of porcine diarrhea often involve mixed infections, which makes diagnosis and treatment more complicated [4]. Early-weaned piglets are particularly susceptible to enteritis due to their underdeveloped immune systems, while pigs in the fattening stage are prone to inflammatory diarrhea following bacterial infections [5]. As the condition progresses, pigs may experience dehydration, diarrhea, and bloody stools, ultimately leading to reduced production performance and substantial economic losses for the farming industry [6]. Currently, antibiotics are the primary clinical treatment for diarrhea [7]; however, due to irregular use, concerns about drug residues and antibiotic resistance have raised safety concerns regarding animal products [8]. The World Health Organization (WHO) has proposed that farmers and the food industry should cease the routine use of antibiotics to promote the growth of healthy animals and prevent diseases [9]. Therefore, the development of new drugs to prevent and treat porcine inflammatory diarrhea as an alternative to antibiotics has emerged as a prominent research area in veterinary clinical studies.

Traditional Chinese medicine (TCM) and its formulations have unique advantages in inhibiting viral replication, anti-inflammatory effects, and antibacterial properties, making them an optimal approach for alleviating inflammation and screening antiviral drugs [10,11]. Lotus leaf is traditionally used to treat spleen deficiency diarrhea, summer dampness diarrhea, and heatstroke symptoms [12,13]. Plant chemical analysis has identified a variety of bioactive components in lotus leaf, including Nuciferine, Quercetin, and Kaempferol [14]. It exhibits a wide range of pharmacological activities, including antiviral, anti-inflammatory, antioxidant, and intestinal microbiota regulation effects [15]. However, the specific mechanisms by which lotus leaf and its active components synergistically combat inflammatory diarrhea remain unclear and require further investigation.

With the continuous development of network pharmacology, database mining of key gene targets for integrated analysis has gained increasing attention from researchers [16]. Based on biomolecular networks, network pharmacology studies the relationship among herbs, components, targets, and diseases from the perspective of interconnection, which meets the needs of systematic treatment of complex diseases [17]. Network pharmacology can also provide a theoretical basis for developing multi-target therapeutic strategies for complex diseases (such as inflammatory diarrhea). For instance, through network pharmacology analysis, Gegen Qinlian Decoction was found to be capable of treating porcine epidemic diarrhea by regulating multiple targets [18].

Therefore, this study intends to analyze the pharmacological action mechanism of lotus leaf by applying network pharmacology and molecular docking. It aims to elucidate the multi-target regulation network of lotus leaf on porcine inflammatory diarrhea, and reveal the interaction of “component–host–disease” of lotus leaf in the prevention and treatment of inflammatory diarrhea. Moreover, it will offer scientific support for promoting the application of natural products in the prevention and control of porcine diseases.

## 2. Materials and Methods

### 2.1. Preparation of Lotus Leaf Extract (LLE)

The method of Yan et al. was used to prepare the LLE [13], and the technical part was improved. Briefly, the dried lotus leaves are crushed through an 80-mesh screen and then soaked in sterile water twice the volume of the lotus leaves for 2 h. The mixture is then boiled and filtered through a 300-mesh screen, a process that is repeated twice. The resulting liquid is then dried with a rotary evaporator (RE-52AA, Shanghai Hefan Instrument Co., LTD., Shanghai, China) to obtain a concentrated paste. Add 70% ethanol equivalent to 5 times the volume of the paste and keep it at 70 °C for 2 h. Finally, the LLE was further dried by rotary evaporator and freeze dryer (CTFD-10S-U, Qingdao Yonghe Chuangxin Electronic Technology Co., LTD., Qingdao, China).

### 2.2. Determination of Components in LLE by LC-MS

To determine the composition of the prepared LLE, the LC-MS method was used to identify the composition; the specific conditions are as follows: UPLC system: C18 column (1.8 μm, 2.1 mm × 50 mm, Agilent Corp, Santa Clara, CA, USA). The mobile phase is 0.1% formic acid water (A) and acetonitrile (B). The gradient elution scheme was 0–1 min, 97%A; 1–12 min, 50%A; 12–15 min, 10%A; 15–17.5 min, 10%A; 17.5–20 min, 97%A. Sample size: 10 μL; column temperature: 40 °C; flow rate: 0.3 mL/min. After elution, composition was performed by an AB Triple TOF X500r system (AB SCIEX, Framingham, MA, USA). The source voltage is 5.5 kV (positive) and −4.5 kV (negative). Source temperature: 550 °C; the pressure of gas 1 and gas 2: 55 psi and 50 psi; the pressure of curtain gas (N2): 25 psi. The maximum allowable error: ±10 ppm. For TOF mass spectrometry acquisition, the scanning range was 100–1000 Da; the accumulation time was 100 ms, DP: 80 V (positive) and −80 V (negative), and for the MS/MS acquisition mode, IDa-based auto-MS2 was performed on the 10 strongest metabolite ions within a full scan cycle (1 s). The *m*/*z* scanning range of the precursor and product ions was 100–1000 Da; the collision energy was 35, and the accumulation time was 100 ms. The *m*/*z* intensity of the target compound was extracted by SCIEX OS software (AB SCIEX, Framingham, MA, USA) and normalized according to a 100% base peak intensity. The primary ion database and ion fragment fitting database provided by software were used to identify the compounds with the highest matching rate (ppm < 10).

### 2.3. Screening of Active Components and Targets of Lotus Leaf

The traditional Chinese medicine database system pharmacology and analysis platform (TCMSP, https://tcmspw.com/tcmsp.php, (accessed on 5 May 2024)) were applied in the prediction of lotus leaf total chemical. The keyword “lotus leaf” was searched, and the components and their corresponding targets were screened according to oral bioavailability (OB) ≥ 30% and drug similarity (DL) ≥ 0.18 [19]. The Uniport database (https://www.uniprot.org, (accessed on 6 May 2024)) was used to standardize the screened targets. The TCMSP database provides the full name of the target acted on by the lotus leaf, which can be matched through Uniport database to obtain the gene symbol.

### 2.4. Screening of Disease Targets for Inflammatory Diarrhea

Using GeneCards database (https://www.genecards.org, (accessed on 1 June 2024)) to search keywords “inflammatory diarrhea in pigs”, and collect the related target genes, merge to heavy, to obtain the pig inflammatory diarrhea disease-related targets.

### 2.5. Diseases and Drugs Share Target Gene Acquisition

The active components of lotus leaf and disease-related targets were integrated to reduce redundancy, and the overlapping genes were identified using the R package 4.0.5 (Venn) to create a Venn diagram illustrating the common targets between the constituents of lotus leaf and the associated diseases.

### 2.6. Lotus Leaf Component—Construction of Target Network of Porcine Inflammatory Diarrhea Disease

The active components derived from lotus leaves, along with the disease targets associated with inflammatory diarrhea, were imported into Cytoscape V3.0 software. These components were organized and arranged based on their degree values, facilitating the identification of drug–target relationships. The drug components and disease-related genes were represented using distinct shapes, thereby enabling the construction of a network that illustrates the relationship between drug components and disease targets. This approach allows for the visualization of the interactions between active pharmaceutical ingredients and their corresponding targets.

### 2.7. Protein–Protein Interaction (PPI) Network and Core Target Screening

The intersection of drug component action targets and disease-related targets was imported into the STRING database (https://cn.string-db.org, (accessed on 10 June 2024)). The species background was designated as “Sus scrofa,” and no interconnected target genes were excluded from the analysis. A confidence threshold of 0.4 was established. Subsequently, a PPI network was generated and analyzed using Cytoscape V3.0 software, with the arrangement based on Degree (degree centrality). The CytoHubba plugin was employed for a comprehensive assessment of various topological features, including Degree (degree centrality), EPC (edge percolation component), NCC (neighborhood component centrality), and BNC (biological network centrality), to identify core targets within the interaction networks.

### 2.8. Gene Ontology (GO) Function and Kyoto Encyclopedia of Genes and Genomes (KEGG) Pathway Enrichment Analysis

To elucidate the potential pathways associated with lotus leaves and porcine inflammatory diarrhea, the DAVID database (https://david.ncifcrf.gov, (accessed on 11 June 2024)) was utilized to GO and KEGG pathway enrichment analyses. Specifically, targets related with lotus leaf and porcine inflammatory diarrhea were input into the DAVID database, with the species background designated as “Sus scrofa”. The GO analysis encompassed three categories: biological process (BP), cellular component (CC), and molecular function (MF). Concurrently, KEGG analysis identified various signaling pathways associated with these targets shared between lotus leaf and porcine inflammatory diarrhea. The enrichment analysis was performed to identify the top ten GO functions with the smallest *p* values, as well as the top fifteen KEGG pathways. Visualization of the results was facilitated through an online bioinformatics mapping platform (http://www.bioinformatics.com.cn, (accessed on 11 June 2024)).

### 2.9. Molecular Docking

To assess the reliability of the network pharmacology analysis, the two-dimensional structures of the active ingredients from lotus leaf were obtained from the PubChem database (https://pubchem.ncbi.nlm.nih.gov, (accessed on 15 June 2024)). Subsequently, the Chem Office 3D V23.0 software was employed for energy minimization, and the resulting structures were saved as SDF files for ligands. The three-dimensional structures of the core proteins were sourced from both the Protein Data Bank (PDB) and the Uniport database (https://www.uniprot.org, (accessed on 15 June 2024)). Utilizing PyMOL V3.1 software, water molecules and ligands were removed, and hydrogen bonds were introduced to enhance the stability of the protein structures. Molecular docking was conducted using Auto Dock Vina V1.2 software, wherein each protein underwent semi-flexible docking with small drug molecules for a total of ten iterations. The combinations of small drug molecules and proteins that exhibited the lowest docking energy (measured in Kcal/mol) were identified. The corresponding binding energies were subsequently represented in a heatmap format and visualized using PyMOL software.

### 2.10. In Vitro Experiments

#### 2.10.1. Cell Maintenance and Cell Viability Assay

The Intestinal Porcine Epithelial Cell line (IPEC-J2, BNCC338252, BeNa Biotechnology Co., Ltd., Beijing, China) was cultured in DME/F-12 medium supplemented with 10% fetal bovine serum (12491015, Thermo Fisher Scientific Inc., Shanghai, China), penicillin (100 units/mL), and streptomycin (0.1 mg/mL) (LifeTechnologies Inc., Carlsbad, CA, USA) at 37 °C in a 5% CO_2_ incubator. LPS (93572-42-0) was purchased from Merck Life Science (Shanghai) Co., Ltd. and was dissolved in double-distilled water (ddH_2_O).

IPEC-J2 cells were seeded in 96-well plates at a density of 1.0 × 10^6^ cells/mL and subsequently treated with various concentrations of LPS (0, 2, 4, 6, 8, 10, 12, 14 µg/mL) for 24 h or lotus extract solutions (5, 10, 20, 40, 60, 80, 100 µg/mL) for 24 h, while the control group received cell culture medium only. Following treatment, 10 µL of Cell Counting Kit 8 (CCK-8, glpbio, Montclair, CA, USA) was added to each well, followed by incubation for 30 min under standard conditions. There were six technical replicates per condition, with three independent biological replicates. The absorbance of each well was measured at 450 nm using a microplate reader to calculate cell viability as follows: percentage of cell viability = A_treatment_/A_control_ × 100% (where A = absorbance).

#### 2.10.2. Flow Cytometry Assay

IPEC-J2 cells were seeded in 6-well plates and subsequently treated with drugs for 24 h following adherence to the plates. The culture medium was aspirated, and the cells were gently washed twice with pre-chilled PBS. Cells were then detached using trypsin without EDTA and the reaction was terminated by adding serum-containing medium. The resulting cell suspension was collected, centrifuged at 1000 rpm for 5 min at 4 °C, and the supernatant was discarded. Cells were resuspended in 100 μL of pre-chilled Binding Buffer, followed by the addition of 5 μL of Annexin V-FITC and gentle mixing. The cells were incubated at room temperature in the dark for 15 min. Subsequently, 5 μL of propidium iodide (PI) was added, and the mixture was incubated for an additional 5 min. Finally, 100 μL of Binding Buffer was added to resuspend the cells. Apoptosis was quantified by flow cytometry (Agilent NovoCyte, Santa Clara, CA, USA) and analyzed using FlowJo 10.0 software.

#### 2.10.3. Western Blot Assay

Protein was extracted from harvested IPEC-J2 cells and quantified through bicinchoninic acid (BCA) analysis (Kangwei Century Biotechnology Co., Jiangsu, China), and the same amount of protein was electrophoresed on a 12% SDS polyacrylamide gel, subsequently transferred onto polyvinylidene fluoride (PVDF) membranes (Cytiva Life Sciences, Marlborough, MA, USA). After blocking at room temperature for 2 h, primary antibodies (TNF-α, 60291-1-Ig, Proteintech, 1:2000; Caspase-3, 66470-2-Ig, Proteintech, 1:1000; Caspase-9, 10380-1-AP, Proteintech, 1:1000; Bcl-2, 26593-1-AP, Proteintech, 1:3000) were added for overnight incubation. The membranes were washed and incubated with secondary antibodies conjugated with horseradish peroxidase (HRP) anti-rabbit IgG (220501, Zenbio, 1:10,000) for 1 h at RT, and exposed under chemiluminescent imaging analysis system (MicroChemi4.2, DNR Bio Imaging Systems, Jerusalem, Isreal). Densitometry analysis was performed by Image J (Version 1.8.0, National Institutes of Health, Bethesda, MD, USA). The experiments were carried out in triplicate.

### 2.11. Statistical Analysis

The formula for all data is the mean ± standard deviation (SD). One-way analysis of variance (ANOVA) was used to compare the values among several groups, and the Tukey test was used to confirm the results. The SPSS ver. 25.0 software was used to complete the statistical analyses. A result of *p* < 0.05 was deemed as statistically significant. GraphPad Prism 8.0 software was used for graphing.

## 3. Results

### 3.1. The LC-MS Detection Results of the LLE

**Figure 1:**
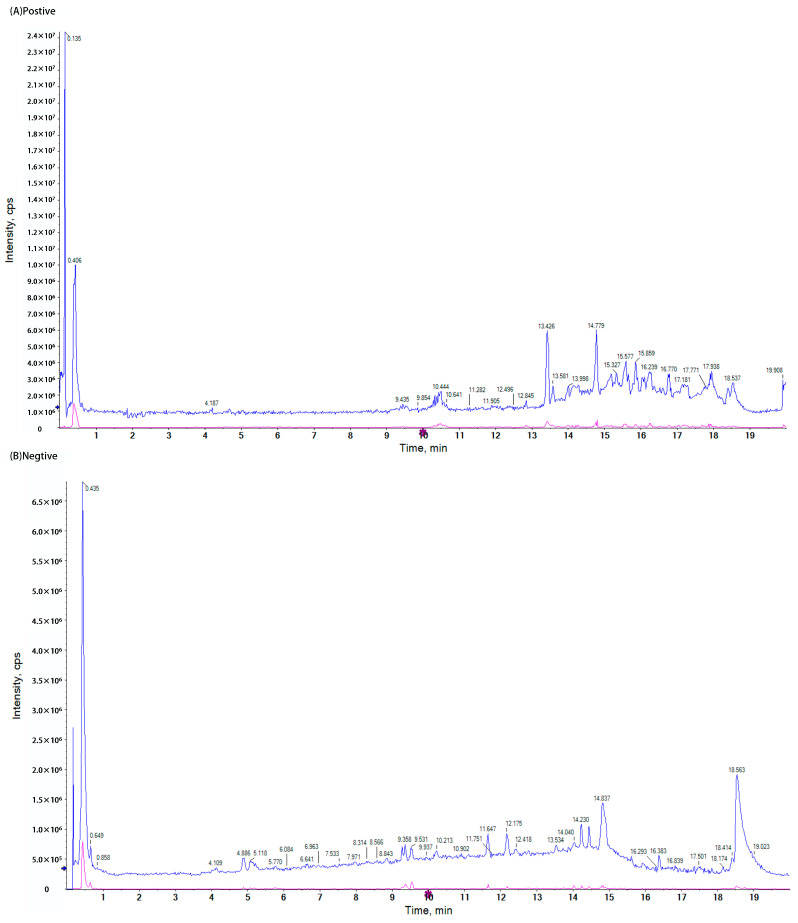
Total ion chromatograms of LLE in both positive and negative ion modes. (**A**) Positive ion mode of LLE, (**B**) negative ion mode of LLE; the abscisa represents time, and the ordinate represents intensity. Scanning range: Blue represents TOF MS *m*/*z* 100 to 1000; red represents TOF MSMS *m*/*z* 100 to 1000.

LC-MS assay was employed to investigate the constituents of LLE. As illustrated in Figure 1, which presented the total ion flow diagram for both positive and negative ion modes, the peak detection times for the positive ion mode predominantly occurred between 10 and 19 min, while the negative ion mode exhibited peak times primarily between 4 and 19 min. The findings are summarized in Table 1, revealing that Quercetin, Nuciferine, Kaempferol, Leucodelphinidin, and Catechin were identified as the compounds with the highest degree of matching within the analyzed range.

### 3.2. Active Components and Targets of Lotus Leaf

In order to avoid all active components of lotus leaves not being fully detected by use of the extraction method and LC-MS conditions, the TCMSP database was used to supplement the active components in lotus leaves, as shown in Table 2. In addition to Kaempferol, Quercetin, and Leucodel-phinidin, more than 10 active components including Sitosterol and Armepavine were found.

### 3.3. Targets of Lotus Leaf Active Components for Inflammatory Diarrhea in Pigs

**Figure 2:**
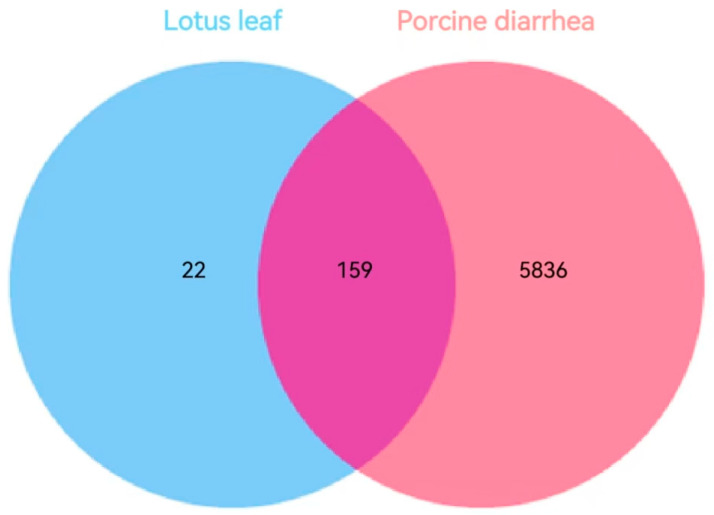
Venn diagram shows the common target of active components of lotus leaf and pig inflammatory diarrhea. The blue section denotes the targets associated with the active components of lotus leaf based on TCMSP, while the pink section indicates the targets related to porcine inflammatory diarrhea based on GeneCards. The overlapping purple region signifies the common targets shared by both categories.

To investigate the relationship between lotus leaf and porcine diarrhea, target genes of both were retrieved from the TCMSP and GeneCards database, and common targets were identified. A total of 181 active components of lotus leaves were identified, while 5995 disease-related targets associated with porcine inflammatory diarrhea were sourced from the GeneCards database. Notably, there are 159 shared targets between these two datasets (Figure 2), which further substantiates the potential applicability of lotus leaf in the treatment of porcine diarrhea-related diseases.

### 3.4. Component–Disease Target Network

**Figure 3:**
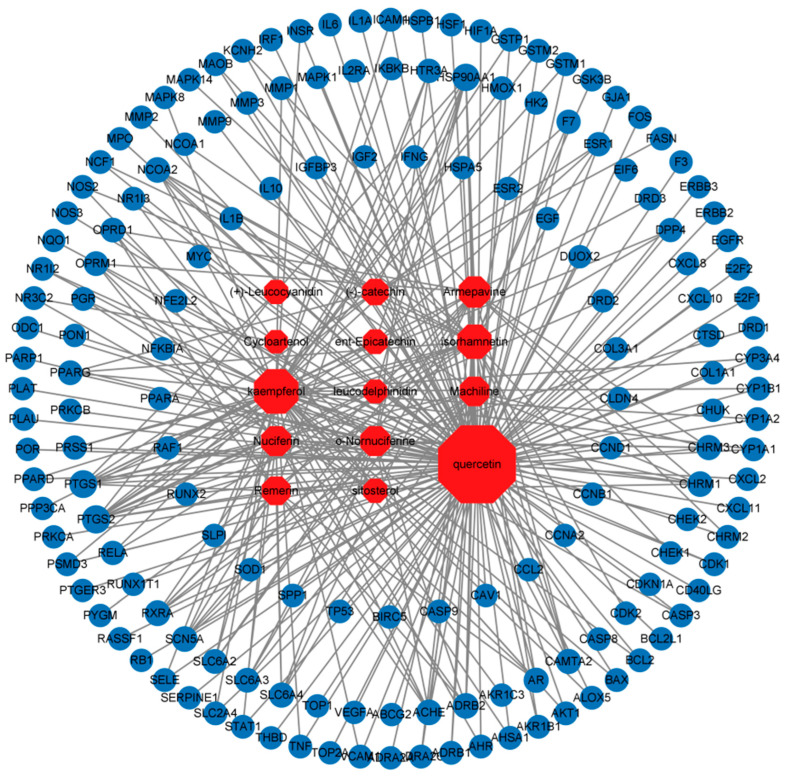
Lotus leaf components—disease target network. Red hexagonal nodes represent active components of lotus leaf, while blue circular nodes represent shared targets related to disease and lotus leaf. Node sizes are determined based on degree centrality, and lines connecting nodes indicate interaction relationships.

The component–disease target network was constructed using Cytoscape 3.0 software, as shown in Figure 3. The results showed that 14 components of lotus leaf potentially acted on 159 disease targets. This indicated that lotus leaf exerts its anti-inflammatory effects through multiple targets and pathways. Key components such as Quercetin, Kaempferol, Nuciferine, and O-nornuciferine interacted significantly with targets such as NF-κB, PPAR, IL-10, TP53, and Caspase-3, suggesting that these are critical targets for lotus leaf in combating inflammatory diarrhea.

### 3.5. Protein–Protein Interaction (PPI) Network Analysis

**Figure 4:**
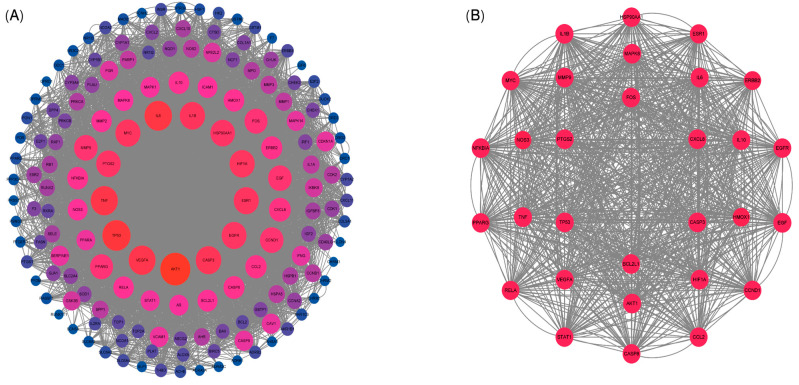
Protein interaction network of common targets of lotus leaf and porcine inflammatory diarrhea. (**A**) Among the 158 protein–protein interaction networks, the larger the node size and the redder the color, the higher the correlation between the proteins; (**B**) a total of 30 core targets were identified with CytoHubba plugin; uniform shape indicates consistent association.

The prediction and construction of the PPI network using the STRING database and Cytoscape software were shown in Figure 4. After excluding unconnected targets, a total of 158 interacting targets were identified (Figure 4A). Furthermore, the results of topological analysis performed using the CytoHubba plugin were presented in Figure 4B, highlighting the identification of 30 core targets. Among these, notable targets included NF-κB, Caspase-8, TP53, AKT, Bcl-2, IL-6, and VEGFA. These findings suggested that these specific target genes might play a significant role in the prevention and treatment of inflammatory diarrhea in pigs, potentially influenced by Lotus Leaf.

### 3.6. GO Function and KEGG Pathway Enrichment Analysis

**Figure 5:**
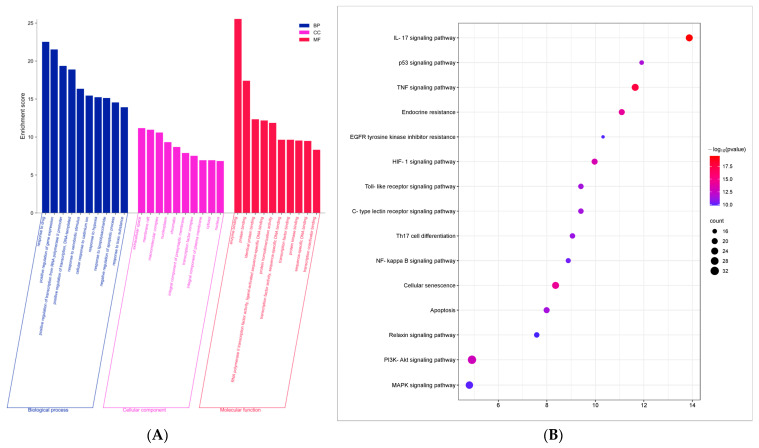
GO and KEGG enrichment analysis. (**A**) GO functional enrichment analysis: blue represents biological processes (BP), purple represents cell components (CC), red represents molecular functions (MF), horizontal coordinate represents different biological functions, and vertical coordinate represents enrichment fraction (−log10 *p* value); (**B**) KEGG enrichment analysis: horizontal coordinate represents enrichment fraction (−log10 *p* value); vertical coordinate is represented as enriched signal pathway; and size of points is determined by number of enriched genes.

The DAVID database (https://david.ncifcrf.gov, accessed on 19 June 2024) was utilized to conduct the GO and KEGG enrichment analysis, and the top 10 GO functions with the smallest *p* values, as well as the top 15 KEGG pathways were shown.The GO analysis (Figure 5A) indicated that the predominant biological processes are associated with drug response and the positive regulation of gene expression. The identified cellular components encompassed extracellular spaces, membrane rafts, and macromolecular complexes. In terms of molecular functions, the analysis highlighted enzyme binding and protein binding activities. Furthermore, the KEGG enrichment analysis (Figure 5B) revealed that the potential therapeutic pathways through which Lotus Leaf may exert effects in the treatment of porcine inflammatory diarrhea include the TNF signaling pathway and apoptosis signaling pathway.

### 3.7. Molecular Docking Results

**Figure 6:**
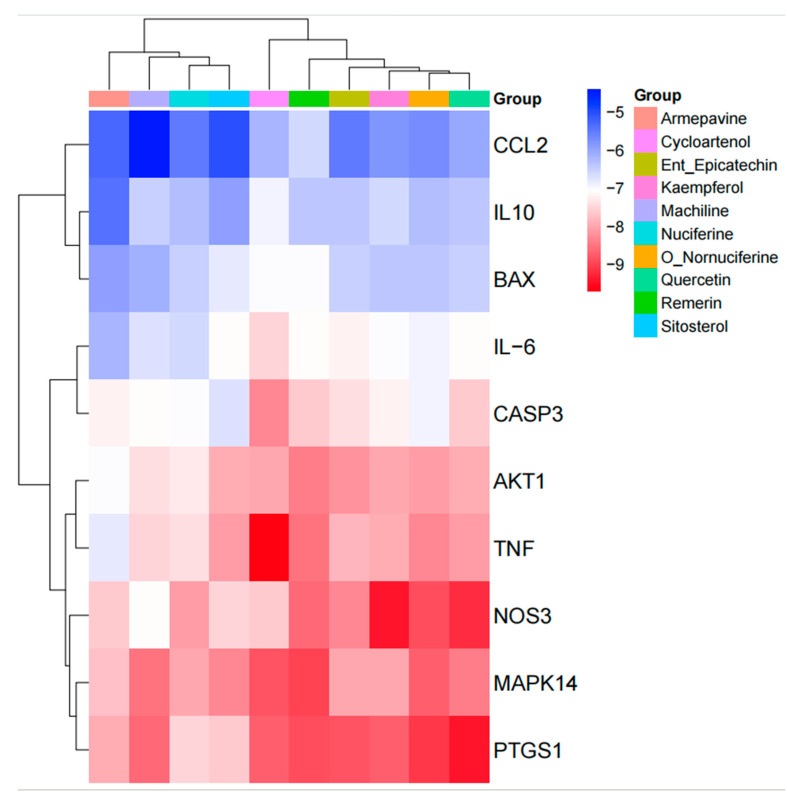
Clustering heat map of molecular docking results. Different small molecular compounds of lotus leaf were marked with different colors in the “group” category, while different protein molecules were positioned along the vertical axis. The binding energy was represented by color gradation: lower binding energy values were indicated by shades closer to blue, signifying stronger binding affinity, whereas higher binding energy values were represented by shades closer to red.

**Figure 7:**
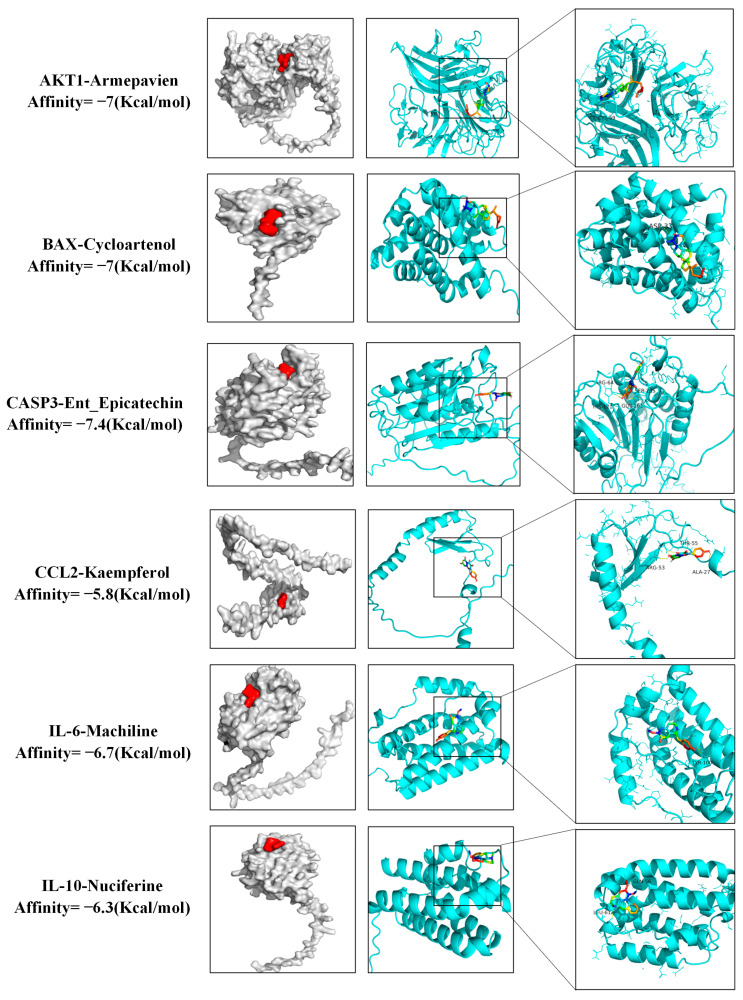
Molecular docking of drug molecules with target proteins. White represents protein and red represents drug small molecule binding patterns, which are specifically represented by binding amino acid residues.

Molecular docking was conducted utilizing AutoDock Vina, with the results presented in the form of a cluster heat map, as illustrated in Figure 6. Notably, the combination of Machiline and CCL2 exhibited a binding energy that was below −5.0 kcal/mol, suggesting that the active constituents of Lotus Leaf possess a relatively stable binding affinity with targets associated with porcine inflammatory diarrhea. Certain docking outcomes were visualized through the use of PyMOL (Figure 7), key targets such as IL-10, IL-6, BAX, Caspase-3, AKT, and TNF demonstrated a strong binding affinity with the drug molecules. Additionally, non-core targets, including CCL2, also displayed favorable binding energy (less than −4.5 kcal/mol) with the components derived from Lotus Leaf.

### 3.8. In Vitro Experiment Results

#### 3.8.1. Effects of LLE and LPS on IPEC-J2 Cell Viability

**Figure 8:**
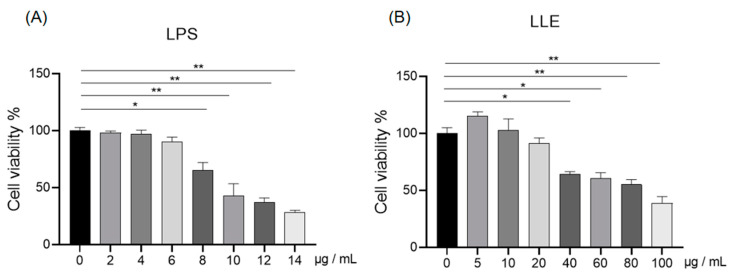
Effects of LLE and LPS on IPEC-J2 cell viability. (**A**) Different concentrations of LPS were applied to IPEC-J2 cells for 24 h; (**B**) different concentrations of LLE were applied to IPEC-J2 cells for 24h. *, *p* < 0.05; **, *p* < 0.01. The data are expressed as the mean ± S.D. (*n* = 6), and differences between mean values were assessed by one-way ANOVA.

As shown in Figure 8A, compared to the cells in control group, the viability of IPEC-J2 cells significantly decreased when the LPS concentration reached 8 µg/mL (*p* < 0.05). Thus, 8 µg/mL was chosen as the concentration of LPS for subsequent experiments. As shown in Figure 8B, compared to the cells in the control group, the viability of IPEC-J2 cells significantly decreased when the concentration of LLE reached 40 µg/mL (*p* < 0.05). However, at concentrations of 20 µg/mL or lower, the cell viability remained unaffected (*p* > 0.05). Therefore, concentrations of 5 µg/mL, 10 µg/mL, and 15 µg/mL LLE were selected for subsequent experiments.

#### 3.8.2. LLE Mitigated LPS-Induced IPEC-J2 Cell Apoptosis

**Figure 9:**
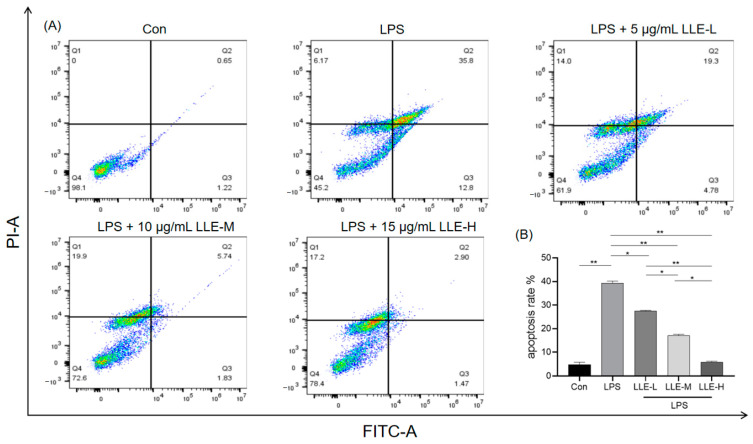
LLE alleviated LPS-induced apoptosis in IPCE-J2 cells. (**A**) Apoptosis detected by Annexin V-FITC/PI stain via flow cytometry; (**B**) apoptosis rate of IPEC-J2 cells. LLE-L: concentration of lotus leaf extract is 5 µg/mL; LLE-M: concentration of lotus leaf extract is 10 µg/mL; LLE-H: concentration of lotus leaf extract is 15 µg/mL. *, *p* < 0.05; **, *p* < 0.01. Data are expressed as mean ± S.D. (*n* = 3), and differences between mean values were assessed by one-way ANOVA.

To further investigate the inhibitory effects of LLE on LPS-induced apoptosis in IPEC-J2 cells, flow cytometry was used to detect the proportion of apoptotic cells. As shown in Figure 9, compared with the control group, the apoptosis rate of cells in the LPS group was significantly increased (*p* < 0.01); compared with the LPS group, the apoptosis rate of cells in the LLE-L group was significantly decreased (*p* < 0.05), and the apoptosis rates of cells in the LLE-M group and LLE-H group were extremely significantly decreased (*p* < 0.01), among which the LLE-H group was significantly lower than the LLE-M group (*p* < 0.05) and extremely significantly lower than the LLE-L group (*p* < 0.01). This indicates that LLE could reverse the apoptosis of IPEC-J2 cells induced by LPS, and the effect of the 15 µg/mL LLE group is better.

#### 3.8.3. LLE Inhibited the Expression Levels of Apoptosis-Related Proteins Induced by LPS

**Figure 10:**
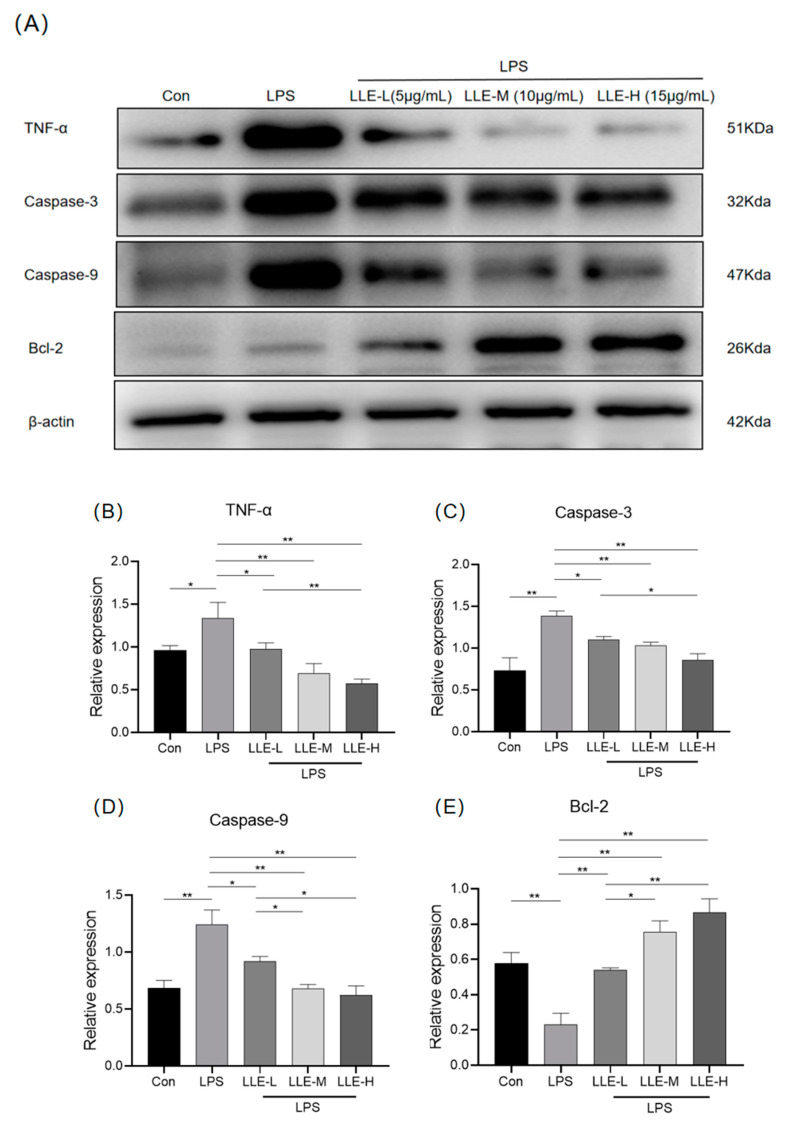
LLE inhibited the expression levels of apoptosis-related proteins induced by LPS. (**A**) expression levels of apoptosis-related proteins were detected using Western blot analysis; the grayscale values of TNF-α (**B**), Caspase-3 (**C**), Caspase-9 (**D**), and Bcl-2 (**E**) were obtained using ImageJ software 2.9.0. LLE-L: The concentration of lotus leaf extract is 5 µg/mL; LLE-M: the concentration of lotus leaf extract is 10 µg/mL; LLE-H: the concentration of the lotus leaf extract is 15 µg/mL. *, *p* < 0.05; **, *p* < 0.01. The data are expressed as the mean ± S.D. (*n* = 3), and differences between mean values were assessed by one-way ANOVA.

As shown in Figure 10, compared with the control group, LPS could significantly up-regulate the expression of TNF-α, Caspase-3 and Caspase-9 proteins (*p* < 0.05), while down-regulating the expression of Bcl-2 (*p* < 0.01). However, different concentrations of LLE under the induction of LPS could significantly inhibit cell apoptosis (*p* < 0.01), promote the expression of Bcl-2, and simultaneously reduce the expression of TNF-α, Caspase-3 and Caspase-9. The results indicated that the LLE could effectively reverse the apoptosis of IPEC-J2 cells induced by LPS. And 15 µg/mL LLE showed the most significant anti-apoptotic effect.

## 4. Discussion

Inflammatory diarrhea in pigs is a common disease in pig farming, significantly affecting pig health and farming efficiency [20]. Farmers often empirically use antibiotics due to difficulties in rapid pathogen differentiation [21,22]. Additionally, antibiotic overuse can lead to environmental contamination and residues in other animals [23,24]. In contrast, traditional Chinese medicine is eco-friendly and sustainable, making it increasingly popular in the livestock industry [25,26]. Lotus leaf is one of the dual-purpose medicinal and edible herbs, and its active components have significant anti-inflammatory and antibacterial effects [13,27].

In this study, network pharmacology was employed to explore the active components of lotus leaf; 15 effective active components were identified. Through a Venn diagram of lotus leaf component targets and animal diarrhea-related targets, 5995 diarrhea-related targets and 181 lotus leaf targets were identified, with 159 common targets. Among these, Nuciferine, Quercetin, and Kaempferol are the key components of lotus leaf in preventing and treating inflammatory diarrhea in pigs. Nuciferine is an aromatic ring-containing alkaloid extracted from lotus leaf. Nuciferine was found to alleviate DSS-induced colitis in mice by inhibiting the release of pro-inflammatory factors such as IL-1 and IL-10 [27,28]. Another study has found that Quercetin could alleviate LPS-induced intestinal inflammation in chickens by inhibiting the TLR4/NF-κB signaling pathway and regulating gut microbial diversity [29]. Quercetin can also reduce the expression of TNF -α and IL-1β and alleviate the colitis in mice induced by DSS [30]. Similarly, in a pig lung injury model induced by H9N2 virus infection, Kaempferol supplementation was found to reduce TNF-α, IL-1β, and IL-6 expression levels while concomitantly enhancing the antioxidant capacity in pigs, with the underlying mechanism involving the inhibition of the MAPK signaling pathway [31]. Supplementation of Kaempferol could down-regulate the secretion of inflammatory factors IL-1β, IL-6 and TNF-α, and regulate the intestinal microbial community [32]. These results indicate that traditional Chinese medicine extracts and their monomers have great preventive and therapeutic effects in inflammatory animal diseases.

The molecular docking method can be used to investigate the interactions between drug compounds and their targets, and to predict their binding modes and affinities. In this study, the molecular docking of the active constituents of LLE with the potential targets for treating porcine inflammatory diarrhea was conducted by computational molecular recognition. However, validation through experimental approaches is also essential; further research will focus on clarifying the most active constituent of LLE and conducting docking studies with porcine proteins.

The pathogenic mechanism of inflammatory diarrhea associated with porcine bacterial enteritis begins with the colonization of pathogens (such as *Salmonella* or *Escherichia coli*) in the intestine, which then invade intestinal epithelial cells through internalization and release LPS [33,34]. LPS activates the Toll-like receptor 4 (TLR4) signaling pathway by binding to TLR4, which recruits MYD88 and promotes NF-κB nuclear translocation, thereby triggering the release of pro-inflammatory cytokines (e.g., IL-6, TNF-α) and chemokines (e.g., IL-8) [35,36]. Additionally, due to endoplasmic reticulum stress, activates Caspases (e.g., Caspase-3/9), causing cell apoptosis [37,38]. Current research has demonstrated that LPS-stimulated models using porcine intestinal epithelial cells (IPEC-J2) or peripheral blood mononuclear cells (PBMC) effectively replicate key phenotypes, including disruption of the intestinal mucosal barrier and abnormal mucus secretion [39,40]. In the present study, LPS stimulation significantly increased both the apoptosis rate and TNF-α expression levels in IPEC-J2 cells, further validating the reliability of this model for simulating porcine intestinal inflammation.

When intracellular inflammatory signaling pathways are activated, pro-inflammatory cytokines such as TNF-α are released, activating Caspase-8 and Caspase-10 through the extrinsic pathway [41]. In the intrinsic pathway, anti-apoptotic Bcl-2 family proteins maintain mitochondrial integrity, while pro-apoptotic Bax translocates to the mitochondria, triggering Cytochrome C release and activation of Caspase-9 and Caspase-3 [42]. Importantly, the anti-apoptotic effects of LLE components corroborate our experimental findings. As an active component of LLE, Quercetin modulates the mitochondrial apoptotic pathway by downregulating Caspase-9, Caspase-3, and Bax protein expression while upregulating Bcl-2 [43]. Kaempferol, another major active component, exerts complementary effects by suppressing Caspase-3 activity through modulation of the MAPK signaling cascade [44]. In the present study, treatment with LLE, particularly at the optimal concentration of 15 μg/mL, significantly attenuated LPS-induced apoptosis in IPEC-J2 cells, as demonstrated by upregulated Bcl-2 expression and downregulated levels of Caspase-3 and Caspase-9. These findings demonstrated that LLE attenuated LPS-induced inflammatory responses in IPCE-J2 cells through modulation of the TNF-mediated apoptotic signaling pathway.

While the in vitro model provides mechanistic insights into LPS-induced inflammation, LPS-induced cellular inflammation cannot accurately represent the actual conditions of porcine diarrhea, as cell cultures lack the systemic interactions and complex tissue-level dynamics present in vivo. Simultaneously, while this study have conducted dose–response studies in vitro, the effective and safe doses of LLE in pigs still require further in vivo studies to confirm its results.

## 5. Conclusions

Network pharmacology analysis revealed that lotus leaf contains 15 active components, among which Kaempferol, Quercetin, and Nuciferine may alleviate inflammatory diarrhea in pigs through signaling pathways such as TNF, PI3K-AKT, and apoptosis. In cell experiments, it was found that LLE could reduce LPS-induced inflammatory responses in IPCE-J2 cells and decrease the apoptosis rate. This mechanism is related with the TNF/Caspase-3 pathway.

## Figures and Tables

**Table 1:** The main components of LLE identified by LC-MS.

Model	Component Name	Component Type	Expected RT	Area	Retention Time	Adduct/Charge	Precursor Mass	Found At Mass	Mass Error (ppm)
positive	Catechin	Quantifiers	3.44	3.15 × 10^5^	3.69	[M + H]^+^	113.963	113.963	3.3
positive	Kaempferol	Quantifiers	5.36	1.99 × 10^5^	5.35	[M + H]^+^	476.305	476.3042	2.5
positive	Nuciferine	Quantifiers	6.94	8.43 × 10^4^	6.95	[M + H]^+^	296.163	296.1633	1
positive	Quercetin	Quantifiers	13.99	4.17 × 10^5^	14	[M + H]^+^	496.337	496.3376	2.6
negative	Leucodelphinidin	Quantifiers	4.15	7.07 × 10^5^	4.13	[M − H]^−^	121.029	121.0292	7.1

Note: Model stands for collection mode; component type: represents qualitative determination method; expected RT: expected retention time; retention time: actual retention time.

**Table 2:** The active ingredients of lotus leaves.

MOL ID	Molecule Name	Oral Bioavailability (%)	Drug-Likeness
MOL000098	Quercetin	46.43	0.28
MOL000354	Isorhamnetin	49.60	0.31
MOL000359	Sitosterol	36.91	0.75
MOL000422	Kaempferol	41.88	0.24
MOL006405	(1S)-1-(4-hydroxybenzyl)-2-methyl-3,4-dihydro-1H-isoquinoline-6,7-diol	67.14	0.23
MOL003578	Cycloartenol	38.69	0.78
MOL007206	Armepavine	69.31	0.29
MOL007207	Machiline	79.64	0.24
MOL007210	o-Nornuciferine	33.52	0.36
MOL007213	Nuciferine	34.43	0.40
MOL007214	(+)-Leucocyanidin	37.61	0.27
MOL007217	Leucodelphinidin	30.02	0.31
MOL007218	Remerin	40.75	0.52
MOL000073	ent-Epicatechin	48.96	0.24
MOL000096	(-)-Catechin	49.68	0.24

## Data Availability

Data will be made available on request.

## References

[1] Liu M., Ma J., Xu J., Huangfu W., Zhang Y., Ali Q., Liu B., Li D., Cui Y., Wang Z. (2024). Fecal microbiota transplantation alleviates intestinal inflammatory diarrhea caused by oxidative stress and pyroptosis via reducing gut microbiota-derived lipopolysaccharides. Int. J. Biol. Macromol..

[2] Yan Z., Wang P., Yang Q., Gao X., Gun S., Huang X. (2023). Change in Long Non-Coding RNA Expression Profile Related to the Antagonistic Effect of Clostridium perfringens Type C on Piglet Spleen. Curr. Issues Mol. Biol..

[3] Zhang J., Yang J., Gao X., Huang X., Luo R., Yang Q., Yan Z., Wang P., Wang W., Xie K. (2022). METTL3 Regulates the Inflammatory Response in CPB2 Toxin-Exposed IPEC-J2 Cells through the TLR2/NF-κB Signaling Pathway. Int. J. Mol. Sci..

[4] Case H.B., Gonzalez S., Gustafson M.E., Dickenson N.E. (2023). Differential regulation of Shigella Spa47 ATPase activity by a native C-terminal product of Spa33. Front. Cell. Infect. Microbiol..

[5] Li L., Li H., Qiu Y., Li J., Zhou Y., Lv M., Xiang H., Bo Z., Shen H., Sun P. (2024). PA-824 inhibits porcine epidemic diarrhea virus infection in vivo and in vitro by inhibiting p53 activation. J. Virol..

[6] Bailey H.M., Fanelli N.S., Campbell J.M., Stein H.H. (2024). Addition of Spray-Dried Plasma in Phase 2 Diets for Weanling Pigs Improves Growth Performance, Reduces Diarrhea Incidence, and Decreases Mucosal Pro-Inflammatory Cytokines. Animals.

[7] Tang Q., Lan T., Zhou C., Gao J., Wu L., Wei H., Li W., Tang Z., Tang W., Diao H. (2024). Nutrition strategies to control post-weaning diarrhea of piglets: From the perspective of feeds. Anim. Nutr..

[8] Trung H.D., Hoang H.V., Thong N.T., Chitana K., Hoai D.T.T., Linh N.Q. (2024). Antibiotics and lectin C for diarrhea control intervention in piglets and influences. AMB Express.

[9] Pillay S., Calderón-Franco D., Urhan A., Abeel T. (2022). Metagenomic-based surveillance systems for antibiotic resistance in non-clinical settings. Front. Microbiol..

[10] Ge F., Yang Y., Bai Z., Si L., Wang X., Yu J., Xiao X., Liu Y., Ren Z. (2023). The role of Traditional Chinese medicine in anti-HBV: Background, progress, and challenges. Chin. Med..

[11] Qiao W.T., Yao X., Lu W.H., Zhang Y.Q., Malhi K.K., Li H.X., Li J.L. (2024). Matrine exhibits antiviral activities against PEDV by directly targeting Spike protein of the virus and inducing apoptosis via the MAPK signaling pathway. Int. J. Biol. Macromol..

[12] Wan Y., Xia J., Xu J.F., Chen L., Yang Y., Wu J.J., Tang F., Ao H., Peng C. (2022). Nuciferine, an active ingredient derived from lotus leaf, lights up the way for the potential treatment of obesity and obesity-related diseases. Pharmacol. Res..

[13] Yan P., Liu J., Huang Y., Li Y., Yu J., Xia J., Liu M., Bai R., Wang N., Guo L. (2023). Lotus leaf extract can attenuate salpingitis in laying hens by inhibiting apoptosis. Poult. Sci..

[14] Tong Y., Li Z., Wu Y., Zhu S., Lu K., He Z. (2021). Lotus leaf extract inhibits ER(−) breast cancer cell migration and metastasis. Nutr. Metab..

[15] He Y., Tao Y., Qiu L., Xu W., Huang X., Wei H., Tao X. (2022). Lotus (*Nelumbo nucifera* Gaertn.) Leaf-Fermentation Supernatant Inhibits Adipogenesis in 3T3-L1 Preadipocytes and Suppresses Obesity in High-Fat Diet-Induced Obese Rats. Nutrients.

[16] Nogales C., Mamdouh Z.M., List M., Kiel C., Casas A.I., Schmidt H. (2022). Network pharmacology: Curing causal mechanisms instead of treating symptoms. Trends Pharmacol. Sci..

[17] Sun Z., Xu Y., An W., Bi S., Xu S., Zhang R., Cong M., Chen S. (2022). Mining Important Herb Combinations of Traditional Chinese Medicine against Hypertension Based on the Symptom-Herb Network Combined with Network Pharmacology. Evid.-Based Complement. Altern. Med. eCAM.

[18] Cui J., Li X., Kang Y., Li P., Guo X., Zhao W., Yang L., Yang Q., Li R., Liu X. (2024). Integrating network pharmacology with pharmacological research to elucidate the mechanism of modified Gegen Qinlian Decoction in treating porcine epidemic diarrhea. Sci. Rep..

[19] Li L., Wang K., Liu Z., Lü Y., Wang C., Yi X., Guo J. (2022). Compound Kushen injection inhibits EMT of gastric cancer cells via the PI3K/AKT pathway. World J. Surg. Oncol..

[20] Yang J., Chen D., Tian G., Mao X., He J., Zheng P., Yu J., Luo Y., Luo J., Huang Z. (2022). 1,25-Dihydroxyvitamin D(3) Negatively Regulates the Inflammatory Response to Porcine Epidemic Diarrhea Virus Infection by Inhibiting NF-κB and JAK/STAT Signaling Pathway in IPEC-J2 Porcine Epithelial Cells. Int. J. Mol. Sci..

[21] Draper L.A., Cotter P.D., Hill C., Ross R.P. (2015). Lantibiotic resistance. Microbiol. Mol. Biol. Rev. MMBR.

[22] Acosta I.C., Alonzo F. (2022). Antibiotic treatment ignites a fire that lasts. Cell Host Microbe.

[23] Fishbein S.R.S., Mahmud B., Dantas G. (2023). Antibiotic perturbations to the gut microbiome. Nat. Rev. Microbiol..

[24] Ngogang M.P., Ernest T., Kariuki J., Mouliom Mouiche M.M., Ngogang J., Wade A., van der Sande M.A.B. (2020). Microbial Contamination of Chicken Litter Manure and Antimicrobial Resistance Threat in an Urban Area Setting in Cameroon. Antibiotics.

[25] Zhang C.C., Wang S., Wang Y.F., Wang H.Y., Qin M., Dai X.Y., Yan B.B., Guo X.Z., Zhou L., Lin H.B. (2023). Application of tissue culture technology of medicinal plants in sustainable development of Chinese medicinal resources. Zhongguo Zhong Yao Za Zhi = Zhongguo Zhongyao Zazhi = China J. Chin. Mater. Medica.

[26] Wang Y., Sheng Z., Li H., Tan X., Liu Y., Zhang W., Ma W., Ma L., Fan Y. (2025). The effects of Fraxini cortex and Andrographis herba on Escherichia coli-induced diarrhea in chicken. Poult. Sci..

[27] Zhu Y., Zhao Q., Huang Q., Li Y., Yu J., Zhang R., Liu J., Yan P., Xia J., Guo L. (2022). Nuciferine Regulates Immune Function and Gut Microbiota in DSS-Induced Ulcerative Colitis. Front. Vet. Sci..

[28] Kulhari U., Kundu S., Mugale M.N., Sahu B.D. (2023). Nuciferine alleviates intestinal inflammation by inhibiting MAPK/NF-κB and NLRP3/Caspase 1 pathways in vivo and in vitro. Int. Immunopharmacol..

[29] Chang Y., Yuan L., Liu J., Muhammad I., Cao C., Shi C., Zhang Y., Li R., Li C., Liu F. (2020). Dihydromyricetin attenuates Escherichia coli lipopolysaccharide-induced ileum injury in chickens by inhibiting NLRP3 inflammasome and TLR4/NF-κB signalling pathway. Vet. Res..

[30] Wang L., Fu R., Meng Y., Liang J., Xue W., Hu H., Meng J., Zhang M. (2024). pH Sensitive Quercetin Nanoparticles Ameliorate DSS-Induced Colitis in Mice by Colon-Specific Delivery. Mol. Nutr. Food Res..

[31] Zhang R., Ai X., Duan Y., Xue M., He W., Wang C., Xu T., Xu M., Liu B., Li C. (2017). Kaempferol ameliorates H9N2 swine influenza virus-induced acute lung injury by inactivation of TLR4/MyD88-mediated NF-κB and MAPK signaling pathways. Biomed. Pharmacother..

[32] Yu R., Zhou Q., Liu T., Liu P., Li H., Bian Y., Liu Z. (2023). Kaempferol relieves the DSS-induced chronic colitis in C57BL/6J mice, alleviates intestinal angiogenesis, and regulates colonic microflora structure. J. Funct. Foods.

[33] Stetsko T. (2022). Bacterial Intestinal Infections of Swine. Sci. Tech. Bull. State Sci. Res. Control Inst. Vet. Med. Prod. Fodd. Addit. Inst. Anim. Biol..

[34] Ruhal R., Kataria R. (2021). Biofilm patterns in gram-positive and gram-negative bacteria. Microbiol. Res..

[35] Wei X., Luo D., Li H., Li Y., Cen S., Huang M., Jiang X., Zhong G., Zeng W. (2024). The roles and potential mechanisms of plant polysaccharides in liver diseases: A review. Front. Pharmacol..

[36] Lu Y.C., Yeh W.C., Ohashi P.S. (2008). LPS/TLR4 signal transduction pathway. Cytokine.

[37] Sun B.H., Zhao X.P., Wang B.J., Yang D.L., Hao L.J. (2000). FADD and TRADD expression and apoptosis in primary hepatocellular carcinoma. World J. Gastroenterol..

[38] Wang Z., Chen X., Liu N., Shi Y., Liu Y., Ouyang L., Tam S., Xiao D., Liu S., Wen F. (2021). A Nuclear Long Non-Coding RNA LINC00618 Accelerates Ferroptosis in a Manner Dependent upon Apoptosis. Mol. Ther. J. Am. Soc. Gene Ther..

[39] Jiao Y., Li H., Ren T., Kim I.H. (2023). Protective effects of methylsulfonylmethane (MSM) on barrier function injury of porcine intestinal epithelial cells (IPEC-J2) induced by lipopolysaccharide (LPS). Can. J. Anim. Sci..

[40] Sadeghi A., Bastin A.R., Ghahremani H., Doustimotlagh A.H. (2020). The effects of rosmarinic acid on oxidative stress parameters and inflammatory cytokines in lipopolysaccharide-induced peripheral blood mononuclear cells. Mol. Biol. Rep..

[41] Guerrache A., Micheau O. (2024). TNF-Related Apoptosis-Inducing Ligand: Non-Apoptotic Signalling. Cells.

[42] Yu W.X., Lu C., Wang B., Ren X.Y., Xu K. (2020). Effects of rapamycin on osteosarcoma cell proliferation and apoptosis by inducing autophagy. Eur. Rev. Med. Pharmacol. Sci..

[43] Yu W., Zhu H., Huang R., Yan B., Xu B., Shi Y., Mao J., Liu Z., Wang J. (2024). Roles of Cyt-c/Caspase-9/Caspase-3/Bax/Bcl-2 pathway in Cd-induced testicular injury in rats and the protective effect of quercetin. Toxicon Off. J. Int. Soc. Toxinol..

[44] Lin C., Wu F., Zheng T., Wang X., Chen Y., Wu X. (2019). Kaempferol attenuates retinal ganglion cell death by suppressing NLRP1/NLRP3 inflammasomes and caspase-8 via JNK and NF-κB pathways in acute glaucoma. Eye.

